# Choline Content of Term and Preterm Infant Formulae Compared to Expressed Breast Milk—How Do We Justify the Discrepancies?

**DOI:** 10.3390/nu12123815

**Published:** 2020-12-13

**Authors:** Anna Shunova, Katrin A. Böckmann, Michaela Minarski, Axel R. Franz, Cornelia Wiechers, Christian F. Poets, Wolfgang Bernhard

**Affiliations:** 1 Department of Neonatology, University Children’s Hospital, Tübingen University Hospital, 72076 Tübingen, Baden-Wuerttemberg, Germany; anna.shunova@med.uni-tuebingen.de (A.S.); katrin.boeckmann@med.uni-tuebingen.de (K.A.B.); michaela.minarski@med.uni-tuebingen.de (M.M.); Axel.Franz@med.uni-tuebingen.de (A.R.F.); cornelia.wiechers@med.uni-tuebingen.de (C.W.); christian-f.poets@med.uni-tuebingen.de (C.F.P.); 2 Center for Pediatric Clinical Studies, University Children’s Hospital, Tübingen University Hospital, 72076 Tübingen, Baden-Wuerttemberg, Germany

**Keywords:** fortification, choline, formula, glycerophosphocholine, maternal milk, phosphatidylcholine, phosphocholine, sphingomyelin, tandem mass spectrometry

## Abstract

Choline/phosphatidylcholine concentrations are tightly regulated in all organs and secretions. During rapid organ growth in the third trimester, choline requirement is particularly high. Adequate choline intake is 17–18 mg/kg/day in term infants, whereas ~50–60 mg/kg/day is required to achieve fetal plasma concentrations in preterm infants. Whereas free choline is supplied via the placenta, other choline carriers characterize enteral feeding. We therefore quantified the concentrations and types of choline carriers and choline-related components in various infant formulae and fortifiers compared to breast milk, and calculated the supply at full feeds (150 mL/kg/day) using tandem mass spectrometry. Choline concentration in formula ranged from values below to far above that of breastmilk. Humana 0-VLB (2015: 60.7 mg/150 mL; 2020: 27.3 mg/150 mL), Aptamil-Prematil (2020: 34.7 mg/150 mL), Aptamil-Prematil HA (2020: 37.6 mg/150 mL) for preterm infants with weights < 1800 g, and Humana 0 (2020: 41.6 mg/150 mL) for those > 1800 g, comprised the highest values in formulae studied. Formulae mostly were rich in free choline or phosphatidylcholine rather than glycerophosphocholine and phosphocholine (predominating in human milk). Most formulae (150 mL/kg/day) do not supply the amounts and physiologic components of choline required to achieve fetal plasma choline concentrations. A revision of choline content in formulae and breast milk fortifiers and a clear declaration of the choline components in formulae is required to enable informed choices.

## 1. Introduction

**Figure 1:**
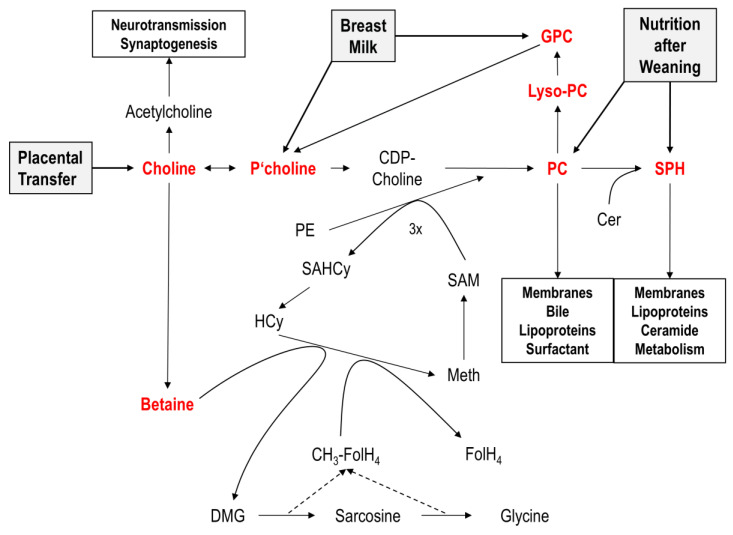
Choline and its individual metabolites in the body and in human nutrition. Graph shows that choline is both substrate for de novo PC synthesis as well as—via betaine—a source of methyl groups/one-carbon units for methionine formation and methylations including endogenous (phosphatidyl)choline (PC) formation. Abbreviations: CDP—choline, cytidylyldiphosphocholine; CH_3_-FolH_4_, methyltetrahydrofolic acid; DMG—dimethylglycine; FolH_4_, tetrahydrofolic acid; GPC—glycerophosphocholine; HCy—homocysteine; Lyso-PC—lysophosphatidylcholine; Meth, methionine; PC—phosphatidylcholine; P’choline, phosphocholine; PE—phosphatidylethanolamine; SAHCy, S-adenosylhomocysteine; SAM—S-adenosylmethionine.

The essential nutrient choline is the head group of phosphatidylcholine (PC) and sphingomyelin (SPH), the main phospholipids of cell membranes in all tissues and of many secretions (surfactant, bile, lipoproteins), which are maintained in tightly regulated concentrations [1,2,3,4,5] (Figure 1). Adequate choline supply is also required for the synthesis of acetylcholine (ACh), a neurotransmitter fundamental to synaptogenesis, development and function of the brain, and leucocyte function [6,7,8]. Plasma choline concentration determines the de novo synthesis of PC and of SPH from (pro-apoptotic) ceramides, where PC is a co-substrate of sphingomyelin synthase (EC 2.7.8.27) [1,9,10]. Increased tissue ceramides are characteristic for pulmonary inflammation and for bronchopulmonary dysplasia, a frequent complication in preterm infants born before the 30-week gestational age. Notably, these patients are characterized by poor intake and low plasma levels of choline [11,12,13,14]. Moreover, its downstream metabolites betaine, dimethylglycine, and sarcosine are methyl donors for the regeneration of methionine from homocysteine. In the form of S-adenosyl methionine (SAM), it is used for the methylation of DNA and histones for epigenetic control and for creatine synthesis. Additionally, betaine is an osmolyte important to kidney function [15,16,17,18].

Choline supply is regarded as being critical in preterm infants, i.e., after untimely cessation of trans-placental supply [2,19,20]. PC and SPH concentrations are high in parenchymal organs, such as the liver, lungs, and brain, so that choline accretion is high during the third trimester and should be high in preterm infants to enable physiologic parenchymal growth [8,16]. Notably, the liver is central to choline homeostasis, accreting PC during choline deficiency from the lungs and other organs via high density lipoproteins [21].

Endogenous synthesis of PC, from which choline can be released, via hepatic phosphatidylethanolamine (PE) methylation by PE-N-methyltransferase (PEMT), is not sufficient for choline requirement and is particularly low in the foetus and preterm infant [22,23]. The adequate choline intake according to the US-Food and Drug Administration and the European Food Safety Authority (EFSA) [24,25] is ~7–8 mg/kg/day for healthy adult humans, substantially higher for pregnant and breast feeding women, and ~18 mg/kg/day for term born infants (0–6 month) [24]. Whereas breast milk supplies term infants with such amounts, its choline content is probably insufficient for the 3–4 fold faster growing preterm infant [16,26,27]. Due to insufficient data, the European Society of the Paediatric Gastroenterology, Hepatology, and Nutrition Committee on Nutrition (ESPGHAN) in 2010 allowed for a higher enteral choline supply of 8–55 mg/kg/day for preterm infants [28]. However, this contrasts data where the daily choline accretion rate required for PC and SPH synthesis alone amounts to ~20 mg/kg/day [2] and intakes of 50–60 mg/kg/day choline equivalent are required to achieve foetal plasma concentrations [22]. However, although infant formulae and breastmilk fortifiers contain choline, there is no standardization with respect to the amount and molecular composition of choline content.

There are different choline-containing compounds characteristic for nutrition during the human life cycle. In utero, free choline is enriched in foetal plasma, resulting in plasma concentrations of 41.4[31.8–51.2] µmol/L, compared to maternal values of 14.1[10.3–16.9] µmol/L. Comparable components for free choline supplementation are choline salts, such as choline chloride or bitartrate. With the start of breast milk feeding (after both preterm and term delivery), however, choline is mainly provided in the form of glycerophosphocholine (GPC) and phosphocholine, with minor contributions of PC, lyso-PC, SPH, and free choline [20,29]. After weaning, PC is the major organic choline component in fresh meat, fish, eggs, legume seeds, nuts, and cereals [30]. Notably, although after birth a significant fraction of absorbed choline is used for the synthesis of methionine from homocysteine requiring betaine, betaine is low in breast milk compared to total choline. Hence, the postnatal increase in plasma betaine originates from choline oxidation and makes exogenous choline the primary source of all methylation processes [22].

The aim of this study was to investigate the concentrations and composition of choline carriers in formulae and multi component breast milk fortifiers for preterm and term infants compared to maternal milk. We analyzed water-soluble (choline, GPC, phosphocholine) and lipidic (PC, lyso-PC, SPH) components, and those of betaine, carnitine, and trimethylamine oxide (TMAO) using tandem mass spectrometry.

## 2. Materials and Methods

### 2.1. Chemicals

Chloroform (HPLC grade) was from Baker (Deventer, The Netherlands). Methanol, acetonitrile, trifluoroethanol, and water (analytical grade) were from Fluka Analytical/Sigma–Aldrich (Munich, Germany). Choline chloride (>99%), phosphocholine, glycerophosphocholine, betaine hydrochloride, *N*,*N*-dimethylglycine were from Sigma–Aldrich (Munich, Germany). D4-choline (Choline-1,1,2,2-d4) chloride as an internal standard was purchased from CDN Isotopes Inc. (Pointe-Claire, QC, Canada). Internal phospholipid standard 1,2-diarachidoyl-sn-glycero-3-phosphocholine (dipalmitoyl-phosphatidylcholine, PC20:0/20:0) was purchased from Avanti Polar Lipids (Alabaster, AL, USA). The purity of the chemicals was checked by liquid chromatography heated electrospray ionization tandem mass spectrometry (LC-H-ESI-MS/MS) (see below). All further chemicals were of analytical grade and from various commercial sources.

### 2.2. Sample Acquisition

The study was carried out in the Neonatal Department of University Children’s Hospital, Tübingen. Two separate breast milk aliquots (0.5 mL), taken at 8–14 h distance, from 21 mothers at postnatal day 8 to 28 after preterm delivery (24–31 completed weeks gestational age) were taken after ethical approval, study registration (ClinicalTrials.gov, Identifier: NCT02509728), and written consent. Sample aliquots were taken from a total breast milk portion after gentle swinging for homogenization using a 500 µL-Eppendorf^®^ pipette, transferred into a 1.5 mL Eppendorf^®^ vial, and stored at −80 °C until analysis. Mean values of the two milk samples of individuals were used for further analyses. Formulae were provided by commercial suppliers in the years 2015–2020 (see Table 1) and stored according to the manufacturers’ instructions until analysis.

### 2.3. Sample Extraction

Milk and formula samples were extracted according to Bligh and Dyer [31]. Glassware for sample extraction was cleaned with methanol to avoid sample contamination with plasticizers or detergents. Multicomponent fortifiers were added to water (instead of expressed breast milk) according to the Manufacturer’s advice for breast milk fortification; formulae were ready-to-use. A 100 µL sample was transferred into a glass vial with a screw cap; 100 µL D4-choline chloride (150 nmol/mL in water), 25 µL PC20:0/20:0 (500 nmol/mL in trifluoroethanol: methanol [2 + 1]) as internal standards, and 5 µL BHT solution (20 mg/mL in ethanol) as an antioxidant were added. Water (0.6 mL), 2.4 mL methanol, and 0.8 mL chloroform were then added and the mixture stirred to achieve a ternary mixture kept at 4 °C for 1 h. Water (1.6 mL) and 2.4 mL chloroform were then added for phase separation; the samples were vigorously stirred and centrifuged at 3000× *g* and 4 °C for 20 min. The upper phase (4.2 mL), containing choline and its water soluble derivatives, and the organic lower phase (adjusted to 4 mL with chloroform: methanol [2:1, *vol*/*vol*]), containing PC, SPH, and lyso-PC, were stored at −80 °C until analysis.

### 2.4. Analysis of Choline, Water Soluble Choline Metabolites and Phospholipids

Analysis was performed as previously described [20,22,26] with minor modifications. The mass spectrometry device imbedded a Finnigan Surveyor Autosampler Plus, a Finnigan Surveyor MS Pump Plus, and a TSQ Quantum Ultra equipped with a heated electrospray ionization interface (H-ESI) (Thermo Fisher Scientific, Dreieich, Germany). Choline and its water-soluble metabolites were analyzed using a HILIC Plus^®^ column (2.1 × 100 mm, 3.5 μm particle size, Agilent Technologies, Böblingen, Germany) at 40 °C. A sample of the upper phase (100 μL) was diluted with 1 mL water and 15 μL injected. The mobile phases were (A) acetonitrile:water:formic acid (90:9.9:0.1, *vol*/*vol*) and (B) water:formic acid (99.9:0.1, *vol*/*vol*). Conditions of elution were: 100% A (0→6 min, 600 µL/min), 20% A (→7 min, 800 µL/min), 100% A (→7.5 min, 800 µL/min), and 100% A (→8,9 min, 800 µL/min). Components were analyzed at positive ionization in the selected reaction monitoring (SRM) mode using mass by charge (*m*/*z*) transitions of 104→60 (choline), 108→60,61 (D4-choline), 118→59 (betaine), 184→86 (phosphorylcholine), 104→58 (dimethylglycine), 258→104 (glycerophosphocholine), and 162→60 (carnitine). Concentrations were calculated from total ion counts relative to the internal standard D4-choline and according to the calibration curves of individual components, as previously described. For lipid analysis, the chloroform phase was diluted by 1:11 with chloroform:methanol (60:40, *vol*/*vol*) and 25 μL injected. PC, lyso-PC, and SPH were separated isocratically using a Polaris 3 Si-A column (2.0 × 100 mm; Agilent Technologies, Böblingen, Germany) at 40 °C, a mobile phase of chloroform:methanol:300 mM ammonium acetate (60:38:2, *vol*/*vol*), and flow adjustment to optimal peak separation and analyte quantification (400 µL/min:0→0.05 min, 600 µL/min:0.06→1.30 min, 400 µL/min:1.40→7.00 min). Individual molecular species were analyzed at positive ionization in the selected reaction monitoring (SRM) mode. Phosphocholine (*m*/*z* = +184) was used as the diagnostic fragment for PC, SPH, and lyso-PC analysis, where individual molecular species were summed up, as previously described [20,22].

### 2.5. Statistics

Statistical analyses were done with JMP 14 and GraphPad Instat3.0. Normal distribution was tested with the Shapiro Wilk test. For breast milk (*N* = 21), the values of two separate samples at 12–16 h distance of 1 day were combined, and means and standard deviation of these 2-sample-means of *N* = 21 individuals are provided. Standard deviation of formulae and fortifiers is provided only in cases where at least three batches of a product were available. Otherwise, only means are indicated. In cases where only one formula/fortifier sample was available, where there were several containers from only a single batch, or there was an availability of only two batches, only mean values are indicated. If at least three batches were available, mean values and standard deviation is provided.

## 3. Results

**Figure 2:**
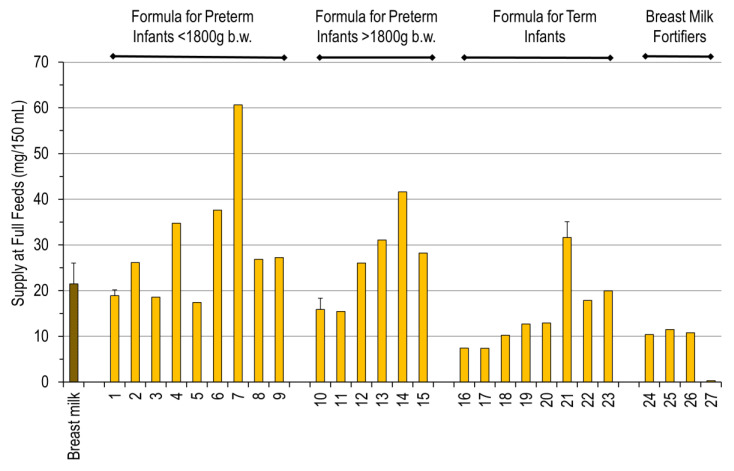
Daily Intake of Total Choline by Infants at Full Feeds. Products are grouped according to the target groups as “Formula for Preterm Infants” below or above 1800 g body weight (b.w.), “Formula for Term Infants”, and “Breast Milk Fortifiers” for preterm infants. Individual product numbering is indicated in Table 1. Data are mean and standard deviations of breast milk from 21 mothers of preterm infants and a convenience sample of formulae from different producers. No standard deviation is provided if there was only one sample, several samples of a single batch, or less than three different batched available for analysis.

Quantitative data are shown as supply in mg/kg/day at an enteral intake of 150 mL/kg/day of breast milk or formula or as the additional supply provided by the breast milk fortifier assuming fortification of 150 mL/kg/day of breast milk according to the manufacturers’ instructions (4–5 g/100 mL). Figure 2 shows the wide range of total choline supply via breast milk, formulae, and breast milk fortifiers. Breast milk supplied 21.5 ± 4.5 mg/150 mL choline. The range of choline supply, relative to breast milk, was 0.9–2.8 fold for preterm infant formula < 1800 g body weight, 0.7–1.9 fold for infants > 1800 g, and 0.3–1.6 fold for term infant formula. Among the products for preterm infant < 1800 g b.w., eight products supplied more choline than breast milk, namely BEBA Frühgeborenennahrung Stufe 1 (2020), Aptamil PREMATIL (2020), PREMATIL HA (2020), and Humana 0-VLB (2015) and medical* (2015). For those >1800 g, Aptamil PDF (2016;2020), Humana 0 (2020), and Humana 0 HA (2020), provided more choline than breast milk. For term infants, most products provided far less choline than preterm infant breast milk, except Aptamil PRE (2020) and Humana Anfangsmilch PRE. The breast milk fortifier BEBA FM 85 (2020) adds another 50–54% of the mean choline content of breast milk, whereas Aptamil FMS does not contribute choline (Figure 1).

**Figure 3:**
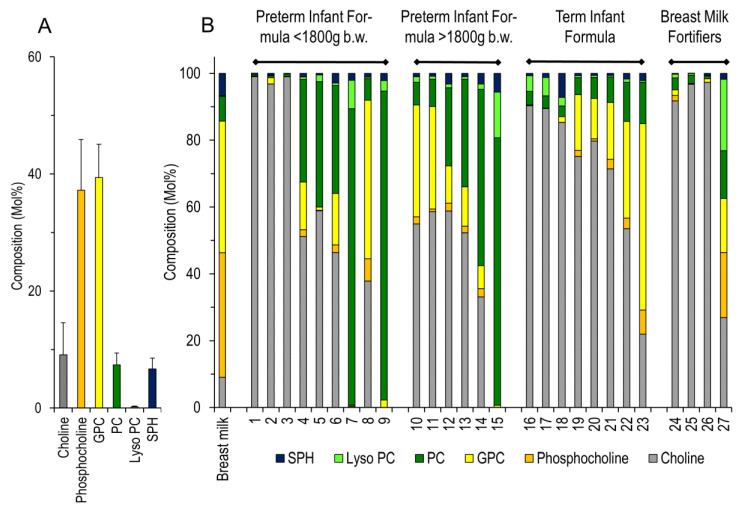
Molar Composition of Preterm Infant Breast Milk. (**A**) Medians and interquartile ranges, and (**B**) Individual components containing a choline moiety in formula/fortifiers compared to breast milk. For details, see the legend to Figure 2. Individual product numbering is indicated in Table 1. Abbreviations: GPC—glycerophosphocholine; PC—phosphatidylcholine; Lyso PC—lyso-phosphatidylcholine; SPH—sphingomyelin.

In breast milk, the primary choline carriers were phosphocholine 37.2 ± 8.6%, glycerophosphocholine (GPC) 39.4 ± 5.7%, whereas free choline 9.1 ± 5.5%, PC 7.4 ± 2.1%, SPH 6.7 ± 1.9% and lyso-PC 0.2 ± 0.1% were minor compounds (Figure 3A). None of the formulae or fortifiers studied reflected the composition of choline carriers in breast milk (Figure 3B). In many products, choline supply was dominated by free choline, with little phosphocholine and glycerophosphocholine. However, there were a few exceptions: Humana 0-VLB and Humana 0 HA for preterm infants < 1800 g and >1800 g, respectively, predominantly comprised PC, and Humana medical* 0 for preterm infants < 1800 g and Humana Anfangsmilch PRE for term born infants, respectively, were enriched in glycerophosphocholine (GPC). Breast milk multicomponent fortifiers provided free choline or no choline at all.

**Figure 4:**
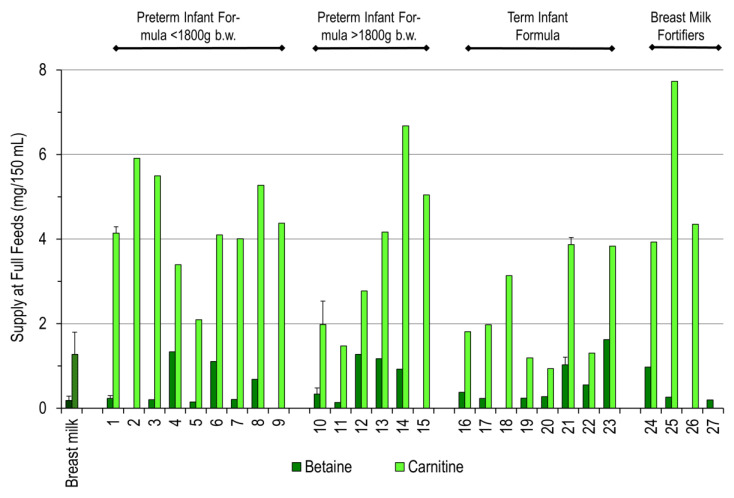
Daily Intake of Choline Derivatives containing a Trimethylated Nitrogen Group. Betaine is a metabolite derived from choline, whereas carnitine requires choline/betaine-derived activated methyl groups for synthesis. For details, see the legend to Figure 2. Individual product numbering is indicated in Table 1. Data are expressed as intake at full feeds. For details, see the legend to Figure 1.

Compared to breast milk, formulae and fortifiers mostly contained significantly more betaine and carnitine (Figure 4). For betaine, this applied to “Aptamil Prematil”, “Aptamil PDF”, and “Aptamil PRE” (2020) as well as “Humana” products. Carnitine content was significantly higher in most products relative to breast milk, except for older (2011–2015) BEBA and Aptamil products for preemies > 1800 g b.w. and term infants.

## 4. Discussion

The importance of choline as an essential nutrient for prenatal development has been known for some time, but only recently has its importance emerged, and higher choline intake during pregnancy as well as after preterm delivery have been suggested [8,13,24,25,27]. There are no firm health claims by the European Food Safety Authority (EFSA) [25], and the ESPGHAN allows for wide ranges of choline supply in preterm infant nutrition (8–55 mg/kg/day) [28]. However, the choline accretion required for the increasing pool sizes of PC and SPH alone, to assure normal fetal growth during the third trimester, is ~20 mg/kg/day, but this is not always provided [2]. Moreover, the rate of PC synthesis for membrane formation and cell growth clearly depends on extracellular (plasma) concentration of choline, as its uptake is mediated by low-affinity transporters [1]. Finally, formation of betaine from choline increases after birth and is higher in preterm infants compared to the fetus, which increases choline requirements [22,26]. Consequently, the amount of choline administration required to achieve plasma choline concentrations in preterm infants equaling those of the age matched fetus suggests that adequate intake is at least in the upper range of the ESPGHAN recommendations or even beyond (probably >/ = 55 mg/kg/day) [2,22,26].

PC and SPH function as structural tissue components, making choline a constitutive rather than a vitamin-like component. Free choline and its water-soluble derivatives phosphocholine, alpha-glycerophosphocholine (GPC), and cytidylyldiphosphocholine (CDP-choline) comprise only 10%, and ACh only 1% of the total choline of an organism’s choline pool. Additionally, after birth, a large proportion of enteral choline supply is oxidized to betaine [22,26]. Consequently, the distribution of choline-derived molecules in the body is complex and dominated by PC, SPH, and betaine. Moreover, PC and SPH are precursors of many tissue hormones and second messengers, such as diacylglycerols, eicosanoids, docosanoids, phosphatidic acid, ceramides, and other sphingolipids, which are all essential in the regulation of organ function, development, termination of inflammation, and repair [12,32,33]. Hence, choline deficiency likely affects tissue development and regulation in many aspects. The Michaelis Constants (KM) of ubiquitous choline transporters (CTL2-4; of 30–100 µmol/L) at the basolateral membranes indicate that choline uptake by cells is proportional to its plasma concentration. The rapid drop of plasma choline after preterm birth, from 41(32–51) µmol/L to 22(16–28) µmol/L will therefore result in decreased cellular uptake, which suggests that current postnatal choline supply is not sufficient in preterm infants and may explain why preterm infants in the 2020s still show inadequate lean body mass growth (if compared to growth in utero). This also applies to preterm infants fed with non-choline supplemented breast milk [22,26].

Breast milk is considered optimal to supply term infants with the required amounts of all nutrients. However, this does not apply to preterm infants whose physiological growth rate is 3–4× higher, therefore requiring higher amounts of macro- and micronutrients. Notably, breast milk of mothers after preterm delivery, as presented in these results, comprises 39% less total choline than term breast milk [29]. By contrast, the molecular composition of choline-containing components (for details see Figure 1) is similar in breast milk after term and preterm birth, and is dominated by the water-soluble esters GPC and phosphocholine, followed by phospholipids (PC, SPH, lyso-PC) and free choline [13,26,29]. Cytidylyldiphosphocholine (CDP-choline) was virtually absent from breast milk, although other pyrimidine components are present in breast milk, and the combination of choline with a pyrimidine might be reasonable as cytosine contributes to choline metabolism [34,35]. The SPH to PC ratio in breast milk was higher than previously reported [29]. Our recent findings may be more reliable because of the separation of these phospholipid classes by liquid chromatography prior to mass spec analysis so that interference of isotopic SPH and PC species was avoided.

Breast milk supplies preterm infants with ~17–25 mg/kg/day choline, which fulfils the suggestions of the IoM/NAM (17–18 mg/kg/day) at 150 mL/kg/day [24]. With such intake, plasma choline in preterm infants is in the range of 23(18–26) µmol/L to 18(16–22) µmol/L. With additional supplementation with 30 mg/kg/day choline chloride for 8–10 day, plasma choline increased to 35(33–42) µmol/L [22,26], similar to fetal plasma choline concentrations. In spite of this, no recommendations for the choline content and molecular composition of preterm and term infant formulae and breast milk fortifiers exist to date.

Regarding the choline concentrations of the different formulae and milk fortifiers, there are substantial differences in the resulting daily total choline supply. Choline contents of older products for preterm infants with birth weights below and above 1800 g birth weight were similar to those of breast milk, being therefore far below the amount we assume to be required (50–60 mg/kg/day) to achieve plasma concentrations of 30–50 µmol/L, as found in age-matched fetuses. Newer formulae, particularly Aptamil Prematil (HA) (<1800 g b.w.) and Humana 0 (>1800 g b.w.) provided 35 to 42 mg/kg/day at intakes of 150 mL/kg/day. For term infant formulae studied herein, even recent products frequently contain less choline than breast milk, and only Aptamil PRE (2020) surmounted the total choline concentrations of breast milk. Among the breast milk fortifiers, BEBA FM 85 (2016, 2019) as well as the Nestlé Study B.M.S. (2014) contributed 10.4–11.5 mg total choline per 150 mL. However, Aptamil FMS (2016) contained virtually no choline. Therefore, the fortifiers studied herein maximally added 50% of the choline being present in breast milk, summing up to 30–35 mg/kg/day of choline equivalent at full feeds, whereas 55 mg/kg/day might be required for near-fetal plasma choline concentrations—and for potentially normal growth and development.

Similarly, the proportions of individual choline carriers are very different between products, and substantially different from breast milk. In no product, except preterm infant formula “Humana Medical 0” from 2015 and term infant formula “Humana Anfangsmilch PRE” from 2019, glycerophosphocholine was a major constituent. Most products are dominated by free choline, PC, or their combination. Only products rich in PC comprise SPH, but at a much lower SPH/PC ratio than that found in breast milk. The relevance of a high SPH/PC-ratio, as was also found in milk fat globule membranes (MFGO) for intestinal epithelia, is under debate [36,37]. As outlined before, the only biological situation where free choline is the dominating compound is intrauterine (parenteral) nutrition, whereas after birth water-soluble and amphiphilic esters dominate GPC, phosphocholine, and PC. There are few data comparing the individual compounds, but those that do point to a better bioavailability and delayed kinetics of GPC and PC compared to free choline [38]. Even more important may be bacterial choline degradation prior to its absorption, leading to trimethylamine (TMA) formation, which is subsequently metabolized to TMA oxide (TMAO) by flavin-containing monooxygenase 3 (FMO3; EC 1.14.13.148) in the liver [39]. In healthy adults, TMAO is not formed from ingested PC, but from free choline provided as choline bitartrate [40]. Hence, the type of choline supplement may define bioavailability. Hepatic expression of FMO3 develops postnatally in term infants [41,42], but its expression in preterm infants is unknown so that TMA may be formed without detection of TMAO in preterm infant plasma.

Hence, PC may be useful to deliver choline by formulae and breast milk fortifiers if the exocrine pancreas and terminal ileum function properly. Whether supply of additional 50–60 mg choline in the form of PC will be effectively assimilated in preterm infants has to be evaluated in clinical trials. Although GPC rather than PC is the major choline carrier in milk, it must be noted that the liver secretes ~50% of the hepatic PC pool per day into the duodenum via bile that has to be recycled effectively after cleavage of PC by pancreatic phospholipase A2 IB (sPLA2IB) and neonatal pancreatic lipase-related protein 2 (PLRP2) [42].

## 5. Conclusions

Total choline concentrations in formulae and breast milk fortifiers for preterm and term infants are highly diverse and frequently do not match breast milk. Only few formulae match the estimated adequate intake of choline for preterm and term infants, whereas no fortifier adds sufficient amounts to achieve the estimated adequate intake for preterm infants in fortified breast milk. Composition of choline carriers in formulae and fortifiers does not match that of breast milk and are frequently composed of either free choline or PC rather than GPC and phosphocholine. A revision of choline content in formulae and breast milk fortifiers and a clear declaration of choline content by choline derivative in products is required to enable informed choices.

## Figures and Tables

**Table 1:** Formulae and breast milk fortifiers for term and preterm infants. Individual compounds were stored according to the manufacturers’ instructions or frozen at −80 °C if not extracted before expiry date. Year of expiry date is indicated at the end of the product name.

**No**	**Full Formula for Preterm Infants below 1800 g Birth Weight**	
1	BEBA Frühgeborenennahrung Stufe 1-2011–2012	Ready to use
2	BEBA Frühgeborenennahrung Stufe 1-2020	Ready to use
3	BEBA Frühgeborenennahrung Stufe 1 PRO HA-2015	Ready to use
4	Aptamil PREMATIL-2020	Ready to use
5	Aptamil PREMATIL HA-2020	Ready to use
6	Aptamil Prematil HA-2015	Ready to use
7	Humana 0-VLB-2015	Ready to use
8	Humana 0 medical-2015	Ready to use
9	Humana 0-VLB-2020	Ready to use
	**Full Formula for Preterm Infants above 1800 g Birth Weight**	
10	BEBA Frühgeborenennahrung Stufe 2-2011–2012	Ready to use
11	BEBA Frühgeborenennahrung Stufe 2 PRO HA-2015	Ready to use
12	Aptamil PDF-2016	Ready to use
13	Aptamil PDF-2020	Ready to use
14	Humana 0-2020	Ready to use
15	Humana 0-HA-2020	Ready to use
	**Full Formula for Term Infants above 2500 g Birth Weight**	
16	BEBA HA Start PRE-2011	Ready to use
17	BEBA HA PRE PRO HA-2015	Ready to use
18	BEBA SUPREME PRE-2020	Ready to use
19	Aptamil PRE-2011	Ready to use
20	Aptamil PRE-2015	Ready to use
21	Aptamil PRE-2020	Ready to use
22	Aptamil GOS/FOS HA Pre-2015	Ready to use
23	Humana Anfangsmilch PRE-2019	Ready to use
	**Breast Milk Fortifiers for Preterm Infants**	
24	BEBA FM 85-2016	5.0% dilution in water
25	Nestlé Study B.M.S.	5.0% dilution in water
26	BEBA FM 85-2019	4.0% dilution in water
27	Aptamil FMS-2016	4.4% dilution in water
